# Differential Detection of Bioavailable Mercury and Cadmium Based on a Robust Dual-Sensing Bacterial Biosensor

**DOI:** 10.3389/fmicb.2022.846524

**Published:** 2022-04-13

**Authors:** Chang-ye Hui, Yan Guo, Han Li, Yu-ting Chen, Juan Yi

**Affiliations:** ^1^Department of Pathology and Toxicology, Shenzhen Prevention and Treatment Center for Occupational Diseases, Shenzhen, China; ^2^National Key Clinical Specialty of Occupational Diseases, Shenzhen Prevention and Treatment Center for Occupational Diseases, Shenzhen, China; ^3^College of Lab Medicine, Hebei North University, Zhangjiakou, China

**Keywords:** whole-cell biosensor, dual-sensing, bioavailability, mercury, cadmium

## Abstract

Genetically programmed biosensors have been widely used to monitor bioavailable heavy metal pollutions in terms of their toxicity to living organisms. Most bacterial biosensors were initially designed to detect specific heavy metals such as mercury and cadmium. However, most available biosensors failed to distinguish cadmium from various heavy metals, especially mercury. Integrating diverse sensing elements into a single genetic construct or a single host strain has been demonstrated to quantify several heavy metals simultaneously. In this study, a dual-sensing construct was assembled by employing mercury-responsive regulator (MerR) and cadmium-responsive regulator (CadR) as the separate sensory elements and enhanced fluorescent protein (eGFP) and mCherry red fluorescent protein (mCherry) as the separate reporters. Compared with two corresponding single-sensing bacterial sensors, the dual-sensing bacterial sensor emitted differential double-color fluorescence upon exposure to 0–40 μM toxic Hg(II) and red fluorescence upon exposure to toxic Cd(II) below 200 μM. Bioavailable Hg(II) could be quantitatively determined using double-color fluorescence within a narrow concentration range (0–5 μM). But bioavailable Cd(II) could be quantitatively measured using red fluorescence over a wide concentration range (0–200 μM). The dual-sensing biosensor was applied to detect bioavailable Hg(II) and Cd(II) simultaneously. Significant higher red fluorescence reflected the predominant pollution of Cd(II), and significant higher green fluorescence suggested the predominant pollution of Hg(II). Our findings show that the synergistic application of various sensory modules contributes to an efficient biological device that responds to concurrent heavy metal pollutants in the environment.

## Introduction

Heavy metals are naturally present in crusts but redistributed in the ecosystem due to natural processes and human activities, especially industrial activities (Rahman and Singh, 2019). Lead (Pb), cadmium (Cd), mercury (Hg), arsenic (As), and chromium (Cr) are non-threshold environmental toxins posing a significant threat to human health, which have received worldwide attention (Balali-Mood et al., 2021). Non-biodegradability and bioaccumulation of heavy metal pollutions have enabled monitor and remediation of environmental heavy metals a long-term process (Hui et al., 2021b; Khan et al., 2021).

Although total element level of heavy metal pollutions could be conveniently determined using continuously developed instrumental analysis methods, speciation analysis of toxic heavy metal mainly depended on the complicated pretreatment methods, which were aimed at purifying and preparing different forms of heavy metal before analysis (Rastegari Mehr et al., 2021; Wang et al., 2021). However, the determination of bioaccessible, bioavailable, and toxic forms of heavy metal pollutions plays a crucial role in predicting their environmental behaviors and ecotoxicological effects (Liu et al., 2017; Chen et al., 2019). The evaluation of bioavailability and bioaccessibility of heavy metal contaminations in the environment mainly depended on biological methods, such as specific heavy metal sensors (Kim et al., 2020). Whole-cell biosensors, stimulating environmental living organisms, can sense and respond to toxic forms of heavy metal contaminations, thereby become ideal choices for monitoring environmental heavy metals (Singh and Kumar, 2021). Prokaryotes are well-known for their exceptional level of adaptation to the surroundings, including the environment seriously polluted by heavy metals. Environmental bacteria have evolved various heavy metal homeostasis systems, which are transcriptionally regulated at the nanomolar level (Jung and Lee, 2019). The MerR family, one of the major metalloregulatory protein families, is involved in bacterial resistance to heavy metals, including Pb, Cd, and Hg. MerR-like regulators have been widely employed to develop specific whole-cell biosensors for monitoring bioavailable heavy metals.

It is well-known that the inherent characteristics of metalloregulator mainly determine the selectivity of genetically programmed bacterial biosensors. However, the biosensor sensitivity is usually determined by genetic circuit engineering and types of reporter genes (Gupta et al., 2019; Hui et al., 2020). Metalloregulator MerR is a Hg(II)-responsive transcriptional factor, which has been employed to develop bacterial whole-cell biosensors using luciferase (Din et al., 2019), β-galactosidase (Hansen and Sorensen, 2000), fluorescence protein (Zhang et al., 2021), and visual pigments (Guo et al., 2021a; Hui et al., 2021a) as the signal outputs. All of these above bacterial biosensors responded to Hg(II) with high selectivity and variable sensitivity. Native Cd(II)-responsive metalloregulator CadR was demonstrated to respond to Cd(II) > Hg(II) >> Zn(II) (Lee et al., 2001; Brocklehurst et al., 2003). Another MerR family member ZntR was found to respond to Zn(II), Cd(II), and Hg(II) (Brocklehurst et al., 1999). Previously developed whole-cell biosensors using these two native metalloregulators as the sensory elements have shown poor selectivity toward heavy metals, including Cd(II), Pb(II), Zn(II), and Hg(II) (Tauriainen et al., 1998; Biran et al., 2000; Riether et al., 2001; Ivask et al., 2004; Cayron et al., 2020; Guo et al., 2021b). A substantial number of studies have been done to improve the performance of whole-cell biosensors for selectively detecting bioavailable Cd(II). The C-terminus of CadR was truncated, and then, the resultant bacterial biosensor responded more selectively to Cd(II) than Hg(II) (Tao et al., 2013). By mutating the metal-binding domain of ZntR, the resultant bacterial biosensor only responded to Cd(II) and Hg(II) (Kang et al., 2018). Cd-specific MerR mutants, generated by directed evolution, have been successfully employed to develop specific Cd(II)-responsive biosensor (Hakkila et al., 2011). A dual-sensing biosensor was assembled by employing CadR and CadC as separate sensory elements, and the dual-sensing genetic circuit facilitated to enhance the selectivity of bacterial biosensor toward Cd(II), Pb(II), and Hg(II) (Hui et al., 2021c).

In summary, these developed MerR-based bacterial biosensors showed a specific response to Hg(II). However, almost all of the available Cd(II)-responsive bacterial biosensors showed a broad response to multiple heavy metals, especially Cd(II) and Hg(II) (Hui et al., 2021b). This study established a dual-sensing biosensor by inserting a MerR-based sensory module and a CadR-based sensory module into a single genetic construct. The resultant whole-cell biosensor showed differential responses to Hg(II) and Cd(II) with double-color fluorescence as the signal output. Our findings show that the dual-sensing biosensor is validated in detecting bioavailable Hg(II) and Cd(II) simultaneously, and the determination of strength ratio of double-color fluorescence can contribute to distinguishing coexisting Hg(II) and Cd(II). It is well-known that various heavy metals usually coexist in polluted environments. We believe that a robust biosensor simultaneously monitoring multiple heavy metal pollutants is valuable, practical, and helpful in real-world applications.

## Materials and Methods

### Bacterial Strains, Plasmids, and Chemicals

All the bacterial strains and plasmids involved in this research are listed in Table 1. *Escherichia coli* (*E. coli*) TOP10 was used as a host strain for cloning and biosensing. Genetically-engineered bacteria were grown at 37*^o^*C in Luria-Bertani (LB) broth containing 1% tryptone (Oxoid, Basingstoke, United Kingdom), 0.5% yeast extract (Oxoid, Basingstoke, United Kingdom), and 1% sodium chloride supplemented with 50 μg/mL ampicillin. DNA primers and fragments were synthesized by Sangon Biotech (Shanghai, China). Restriction endonucleases, reagents, and buffers used in molecular cloning were purchased from Sangon Biotech. All other chemicals were of analytical grade and were obtained from Sigma-Aldrich (St Louis, MO, United States). Stock solutions of cadmium chloride, mercuric chloride, lead nitrate, and zinc sulfate were freshly prepared using ultrapure water and filtered through a 0.22 μM filter. All recombinant plasmids were identified by colony PCR, restriction enzyme digestion, and DNA sequencing.

**TABLE 1:** Bacterial strain and plasmids used in this study.

Strain and vectors	Genotypes or description	References
Strain		
*E. coli* TOP10	F^–^, Φ80*lac*ZΔM15, Δ*lac*X74, *rec*A1	Invitrogen
Plasmids		
pET-21a	Amp*^R^*, T7 promoter, lac operator	Novagen
pT-RFP	T vector carrying *mcherry*	Hui et al., 2018c
pT-GFP	T vector carrying *egfp*	Hui et al., 2018c
pPmer	pET-21a derivative containing *merR* and P*mer* divergent promoter region cloned into *Bgl*II and *Xba*I sites	Hui et al., 2021c
pPcad	pET-21a derivative containing *cadR* and P*cad* divergent promoter region cloned into *Bgl*II and *Xba*I sites	Hui et al., 2021c
pPmer-G	pPmer derivative carrying promoterless *egfp* and *rrn*B terminator cloned into *Nde*I and *Hin*dIII sites	This study
pR-Pmer-G	pPmer-G derivative, a promoterless *mcherry* cassette fused to the downstream of *merR*	This study
pPcad-R	pPcad derivative carrying promoterless *mcherry* and *rrn*B terminator cloned into *Nde*I and *Xho*I sites	This study
pG-Pcad-R	pPcad-R derivative, a promoterless *egfp* cassette fused to the downstream of *cadR*	This study
pR-Pcad	pET-21a derivative containing *cadR*-P*cad-mcherry* cloned into *Bam*HI and *Bgl*II sites	This study
pR-Pcad-Pmer-G	pR-Pcad derivative containing *merR*-P*mer-egfp* cloned into *Bam*HI and *Hin*dIII sites	This study

### Genetic Assembly of Single-Sensing and Dual-Sensing Constructs

**FIGURE 1:**
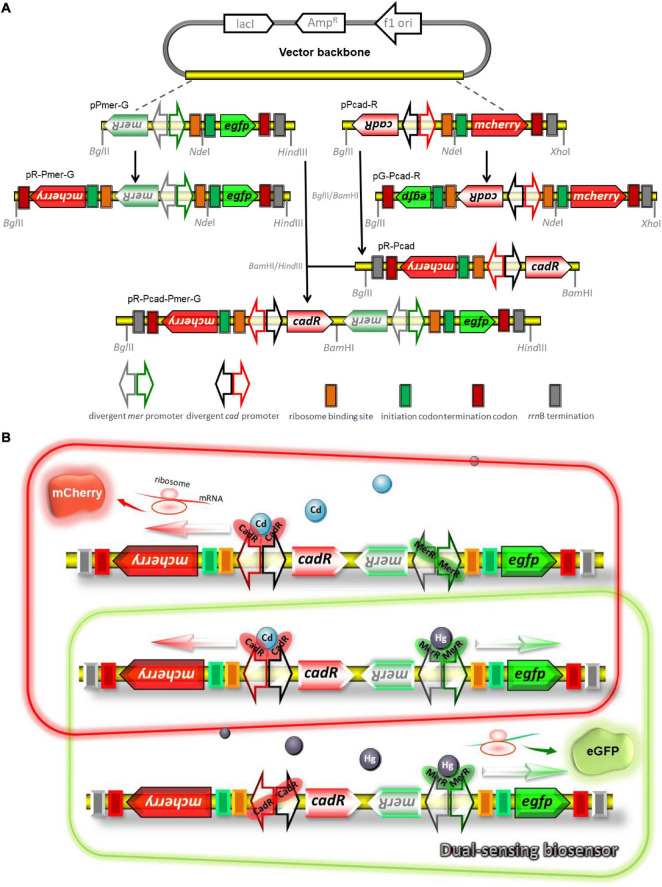
Genetic organization of dual-sensing biosensor for detecting bioavailable Hg(II) and Cd(II). **(A)** Schematic representation of plasmid constructs used in the study. Two kinds of fluorescent reporter modules were placed under the control of Hg(II) sensory element or Cd(II) sensory element to assemble various single-sensing constructs. The Cd(II) biosensing system and the Hg(II) biosensing system were integrated into one genetic construct in two different directions. The DNA sequence and biological annotation of inserted fragments are shown in Supplementary Figure 1. **(B)** Molecular mechanisms of fluorometric determination of bioavailable cadmium and mercury. Dimeric metalloregulator CadR bound to the divergent *cad* promoter activates expression of the red fluorescence protein mCherry in the presence of Cd(II), and dimeric metalloregulator MerR acts as a transcription repressor without Hg(II) exposure (schematic in red box). Dimeric metalloregulator MerR bound to the divergent *mer* promoter activates production of the green fluorescence protein eGFP in the presence of Hg(II) (schematic in green box). When Cd(II) and Hg(II) appear in the cytoplasm simultaneously, the dual-sensing biosensor can differentially detect Cd(II) and Hg(II) with double-color fluorescence output (overlap between the red and green boxes).

The strategy used for the assembly of biosensing constructs is shown in Figure 1A. Recombinant plasmids were assembled by a combination of PCR and subcloning techniques. An 887 bp fragment containing the promoterless *egfp* gene and *rrn*B terminator was PCR amplified from the pT-GFP vector and subcloned into the pPmer vector using the *Nde*I and *Hin*dIII restriction sites to generate pPmer-G. Similarly, an 878 bp fragment containing the promoterless *mcherry* gene and *rrn*B terminator was PCR amplified from the pT-RFP vector and subcloned into the pPcad vector using the *Nde*I and *Xho*I restriction sites to produce pPcad-R. A DNA fragment containing a mCherry open reading frame (ORF) was amplified from pT-RFP and fused with the Hg(II) sensory element *merR*-P*mer* by an overlapping extension PCR as described previously (Guo et al., 2021a) to generate pR-Pmer-G.

Similarly, a DNA fragment containing an eGFP ORF was amplified from pT-GFP and fused with the Cd(II) sensory element *cadR*-P*cad* by an overlapping extension PCR to generate pG-Pcad-R. The *cadR*-P*cad-mcherry* cassette was PCR amplified from pPcad-R and reversely inserted into the *Bgl*II and *Bam*HI sites of pET-21a to generate pR-Pcad. The *merR*-P*mer-egfp* cassette was PCR amplified from pPmer-G and inserted into the *Bam*HI and *Hin*dIII sites of pR-Pcad to generate pR-Pcad-Pmer-G, which was designed as a dual-sensing construct to detect bioavailable Cd(II) and Hg(II) simultaneously. All the biosensing constructs were used for transformation into *E. coli* TOP10 competent cells and bacterial transformants harboring the biosensing constructs were selected on LB agar plates containing 50 μg/mL ampicillin.

A potential mechanism for simultaneous differential detection of bioavailable Cd(II) and Hg(II) based on a dual-sensing system is shown in Figure 1B. When only Cd(II) exists in bacterial cytoplasm, selective binding with dimeric CadR is mediated by the cooperation of thiolate-rich and histidine-rich sites, leading to the conformational change of dimeric CadR (Liu et al., 2019). The resultant activated dimeric CadR is critical for enhancing the transcriptional activity of downstream red fluorescent reporter. Although some studies have shown dimeric CadR could also be activated by intracellular Hg(II) (Tao et al., 2013; Hui et al., 2021c), dimeric MerR will undoubtedly become the dominant transcription activator to trigger the transcription of green fluorescence protein upon exposure to only Hg(II). Both red and green fluorescence will be emitted simultaneously when Cd(II) and Hg(II) coexists.

### Comparison of Responses From Different Single-Sensing Constructs

Single colonies of various single-sensing whole-cell biosensors were picked from the agar plates and used to inoculate 3 mL of LB medium and cultured at 37*^o^*C for 12 h. Overnight cultures were inoculated into fresh LB medium at 1% inoculation amount. To investigate the bidirectional transcription levels of TOP10/pR-Pmer-G and TOP10/pG-Pcad-R upon exposure to their cognate metal ions, TOP10/pR-Pmer-G in lag phase was directly exposed to 0-10 μM Hg(II), and TOP10/pG-Pcad-R was exposed to 0–100 μM Cd(II). To investigate the influence of the cloning direction on the response of the Cd(II)-sensing module, TOP10/pPcad-R, and TOP10/pR-Pcad were exposed to 0–100 μM Cd(II). After culturing at 37*^o^*C for 8 h, bacterial cell density and fluorescent signals were measured.

### Selectivity Test

Overnight cultures of single-sensing TOP10/pPmer-G, single-sensing TOP10/pR-Pcad, and dual-sensing TOP10/pR-Pcad-Pmer-G were inoculated into fresh LB medium at 1% inoculation amount. Stock solutions of Zn(II), Pb(II), and Cd(II) were added at a final concentration of 5, 25, 125, or 250 μM, and stock solution of Hg(II) was added at a final concentration of 5 or 25 μM. After culturing at 37*^o^*C for 8 h, bacterial cell density and fluorescent signals were measured.

### Differential Responses of Whole-Cell Biosensors Toward Hg(II)

Overnight cultures of single-sensing TOP10/pPmer-G, single-sensing TOP10/pR-Pcad, and dual-sensing TOP10/pR-Pcad-Pmer-G were inoculated into fresh LB medium at 1% inoculation amount. A double dilution method described previously (Hui et al., 2021c) was used to obtain 40, 20, 10, 5, 2.5, 1.25, 0.625, 0.3125, 0.156, 0.078, 0.039, 0.0195, 0.0098, 0.0049, 0.0024, and 0 μM Hg(II) exposure groups. The resultant cultures were grown at 37*^o^*C for 8 h before measuring bacterial cell density and fluorescent signals.

### Differential Responses of Whole-Cell Biosensors Toward Cd(II)

Overnight cultures of single-sensing TOP10/pR-Pcad and dual-sensing TOP10/pR-Pcad-Pmer-G were inoculated into fresh LB medium at a 1% inoculation amount. A double dilution method was used to obtain 800, 400, 200, 100, 50, 25, 12.5, 6.25, 3.125, 1.56, 0.78, 0.39, 0.195, 0.098, 0.049, 0.024, 0.012, and 0 μM Cd(II) exposure groups. The resultant cultures were grown at 37*^o^*C for 8 h before assessing the fluorescent signals and bacterial cell density.

### Differential Responses of Dual-Sensing Biosensor Toward Hg(II) and Cd(II)

To study the influence of Cd(II) on the response of the dual-sensing TOP10/pR-Pcad-Pmer-G toward Hg(II), the mixtures of 2.5 μM Hg(II) with 0–400 μM Cd(II) were added into the cultures of TOP10/pR-Pcad-Pmer-G during the lag phase. Double-color fluorescent signals and bacterial density were determined after an 8-h induction at 37*^o^*C.

To evaluate the influence of Hg(II) on the response of the dual-sensing TOP10/pR-Pcad-Pmer-G toward Cd(II), the mixtures of 100 μM Cd(II) with 0–10 μM Hg(II) were added into the cultures of TOP10/pR-Pcad-Pmer-G during the lag phase. The fluorescent signals and bacterial density were measured after an 8-h induction at 37*^o^*C.

### Measurements of Bacterial Density and Fluorescent Signals

Aliquots (100 μL) of bacterial culture were transferred into a 96-well microplate, and bacterial cell density was determined by measuring optical density at 600 nm using a microplate reader (BioTek Epoch, Winooski, VT, United States). Both green fluorescence and red fluorescence emitted from whole-cell biosensors were quantitated using a fluorescence spectrometer (Thermo, Waltham, MA, United States) as previously described (Hui et al., 2018c, 2021c). Briefly, bacterial cultures were diluted to 3 ml with purified water and pipetted into a low fluorescence background quartz cuvette. The intensity of emitted eGFP fluorescence was recorded at 507 nm with excitation at 488 nm, and the intensity of emitted mCherry fluorescence was recorded at 610 nm with excitation at 587 nm. The fluorescent signal is indicated as a fluorescence count value (unit = cnt) and finally calculated as relative fluorescent units (RFU) per OD_600_.

### Microscopic Analysis of Whole-Cell Biosensors

Aliquots (50 μL) of bacterial cultures were spread onto glass slides. After being air-dried at room temperature, the slides were gently washed with purified water to remove the residues of culture ingredients. Bacterial cells were fixed on the glass slides, and then visible, and fluorescent images were taken under 400 × magnification with an Eclipse Ni fluorescence microscope (Nikon, Tokyo, Japan) equipped with a FITC and Texas Red filter as described previously (Hui et al., 2018a,b).

## Results

### Response Characteristics of Single-Sensing Biosensors Assembled With Different Genetic Strategies

To investigate the differential activation of the divergent *mer* promoter, recombinant TOP10 harboring pR-Pmer-G was exposed to 0, 1.25, 2.5, 5, and 10 μM Hg(II). Bacterial density and double-color fluorescence were determined after 8-h incubation at 37*^o^*C. As shown in Supplementary Figure 2A, the green fluorescence continuously increased with Hg(II) exposure at the concentrations range of 0–10 μM. However, the intensity of red fluorescence hardly changed. Thus it can be seen that the transcription of *merR* and its downstream *mcherry* in the opposite direction is not improved upon exposure to Hg(II). Similarly, to study the differential activation of the divergent *cad* promoter, TOP10/pG-Pcad-R was exposed to 0, 12.5, 25, 50, and 100 μM Cd(II). Bacterial density and double-color fluorescence were measured after 8-h incubation at 37*^o^*C. As shown in Supplementary Figure 2B, significantly enhanced red fluorescence was observed with 0–100 μM Cd(II) exposure, and the change of the green fluorescence is unobvious. We can draw a conclusion that the transcription of *cadR* and its downstream *egfp* in the opposite direction is also not improved upon exposure to Cd(II). Single directional transcriptional activation contributes to the subsequent development of the dual-sensing construct.

To investigate the influence of insertion direction on the behaviors of biosensing constructs, TOP10/pPcad-R and TOP10/pR-Pcad in lag phase were exposed to 0, 12.5, 25, 50, and 100 μM Cd(II). The RFU was determined after 8-h incubation at 37*^o^*C (Supplementary Figure 3). As expected, the red fluorescence in both groups increased with 0–100 μM Cd(II) induction. Notably, the responses of two biosensors exposed to the same Cd(II) concentration were similar.

### Heavy-Metal Specificity of Various Whole-Cell Biosensors

**FIGURE 2:**
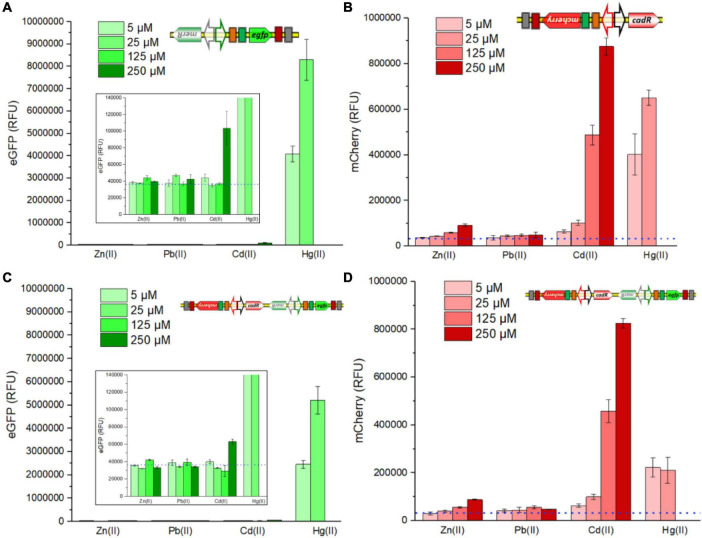
Responses of single-sensing and dual-sensing whole-cell biosensors toward different metal ions. Single-sensing TOP10/pPmer-G, single-sensing TOP10/pR-Pcad, and dual-sensing TOP10/pR-Pcad-Pmer-G in lag phase were exposed to four different metal ions. After incubation at 37*^o^*C for 8 h, green fluorescence derived from TOP10/pPmer-G **(A)**, red fluorescence derived from TOP10/pR-Pcad **(B)**, green fluorescence derived from TOP10/pR-Pcad-Pmer-G **(C)**, and red fluorescence derived from TOP10/pR-Pcad-Pmer-G **(D)** were all determined. The dotted lines show the basal level of double-color fluorescence in whole-cell biosensors without heavy metal exposure (background). The insets prominently show the basal level of green fluorescence in a low range of RFU. Fluorescence intensity values were normalized using the absorbance at 600 nm. Data represent the mean ± SD of at least three independent experiments.

Single-sensing TOP10/pPmer-G, single-sensing TOP10/pR-Pcad, and dual-sensing TOP10/pR-Pcad-Pmer-G were exposed to Zn(II), Pb(II), and Cd(II) at the concentration of 0, 5, 25, 125, or 250 μM, to Hg(II) at the concentration of 0, 5, or 25 μM. The RFU were determined after culturing at 37*^o^*C for 8 h. Largely due to the high specificity of metalloregulator MerR, the green fluorescence, which is inducibly expressed from the *merR*-P*mer-egfp* genetic cassette, selectively responded to Hg(II) in either a single-sensing construct (Figure 2A) or a dual-sensing construct (Figure 2C). A slightly enhanced response toward 250 μM Cd(II) was observed. The red fluorescent signal, which is induced from the *cadR*-P*cad-mcherry* genetic cassette, strongly responded to Cd(II) in a dose-response relationship in either a single-sensing construct (Figure 2B) or a dual-sensing construct (Figure 2D). Furthermore, a strong red fluorescence responsive to Hg(II) was found in the single-sensing construct (Figure 2B), However, the red fluorescence responsive to Hg(II) was significantly decreased in the dual-sensing construct (Figure 2D).

### Responses of Whole-Cell Biosensors Toward Hg(II)

**FIGURE 3:**
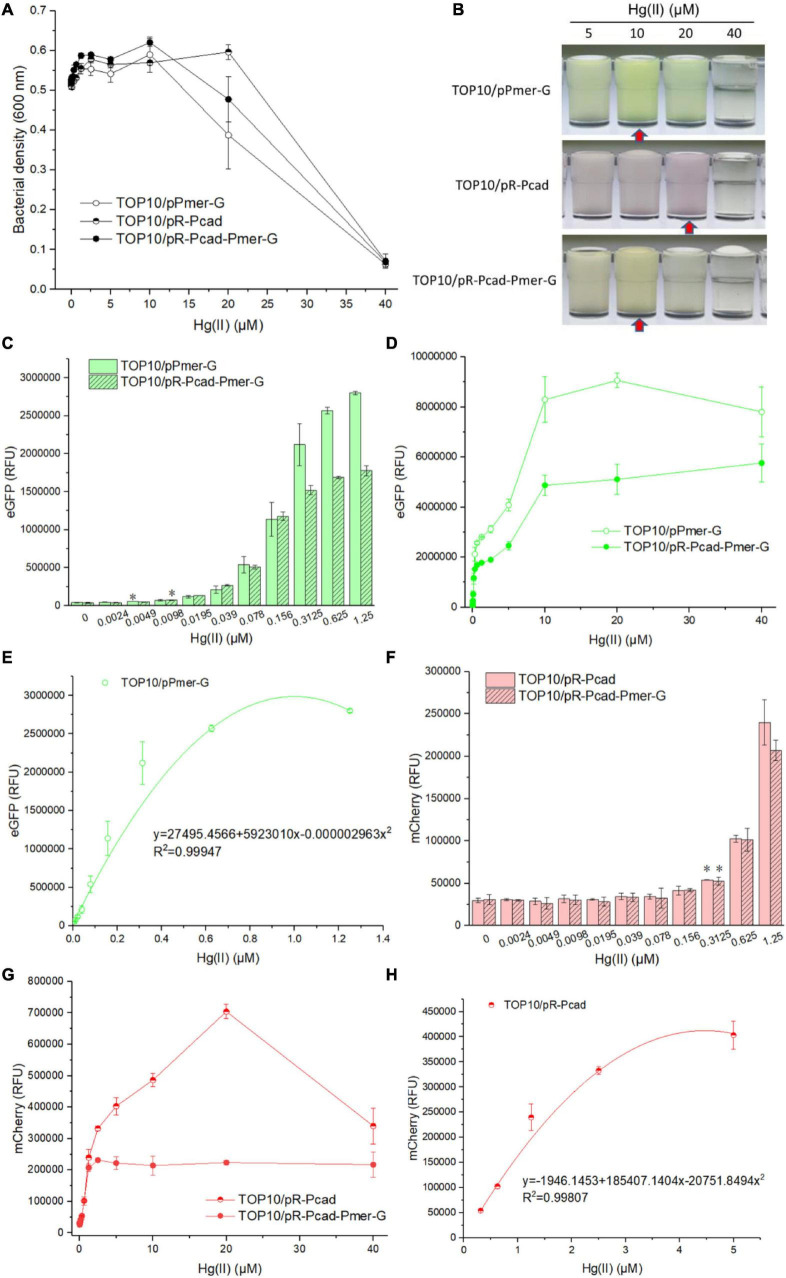
Performance of single-sensing and dual-sensing whole-cell biosensors exposed to increased concentrations of Hg(II). Single-sensing TOP10/pPmer-G, single-sensing TOP10/pR-Pcad, and dual-sensing TOP10/pR-Pcad-Pmer-G in lag phase were exposed to increased concentrations of Hg(II). After incubation at 37*^o^*C for 8 h, bacterial cell densities **(A)** were determined immediately. A representative photo of induced culture **(B)** was shown, and the cultures with high absorbance at 600 nm were marked with red arrow. **(C)** The detection sensitivity when using green fluorescence as signal output. The asterisk represents a statistically significant difference (two-tailed *t* test, *P* < 0.05) compared with the same engineered biosensor not upon exposure to Hg(II). **(D)** The dose-dependent green fluorescent response curve for Hg(II) exposure ranges from 0 to 40 μM. **(E)** Regression analysis of the relationship between relative green fluorescent intensity derived from TOP10/pPmer-G and Hg(II) concentration. **(F)** The detection sensitivity with red fluorescence as signal output. The asterisk represents a statistically significant difference (two-tailed *t*-test, *P* < 0.05) compared with the same engineered biosensor with no Hg(II) exposure. **(G)** The dose-dependent red fluorescent response curve for Hg(II) exposure ranges from 0 to 40 μM. **(H)** Regression analysis of the relationship between relative red fluorescent intensity derived from TOP10/pR-Pcad and Hg(II) concentration. Data represent the mean ± SD of at least three independent experiments.

Single-sensing TOP10/pPmer-G, single-sensing TOP10/pR-Pcad, and dual-sensing TOP10/pR-Pcad-Pmer-G were exposed to 0, 0.0024, 0.0049, 0.0098, 0.0195, 0.039, 0.078, 0.156, 0.3125, 0.625, 1.25, 2.5, 5, 10, 20, and 40 μM Hg(II). Bacterial density and double-color fluorescence were measured after culturing at 37*^o^*C for 8 h. Bacterial densities of three whole-cell biosensors significantly decreased with above 10 μM Hg(II) exposure in two single-sensing biosensors (Figure 3A), probably due to the toxicity of high concentration of Hg(II). Overexpressed double-color fluorescent proteins made the Hg(II)-induced culture color green in TOP10/pPmer-G, red in TOP10/pR-Pcad, and yellow in TOP10/pR-Pcad-Pmer-G (Figure 3B). These colors, which could be easily recognized by the naked eye over a range of Hg(II) from 5 to 20 μM, inevitably interfered with the measurement of bacterial density at 600 nm. Due to the high absorbance of red color at 600 nm, the cultures with overexpressed mCherry always exhibited a high OD_600_ value (Figure 3A). Single-sensing TOP10/pPmer-G with green fluorescence as the unique signal could detect as low as 0.0049 μM Hg(II), and dual-sensing TOP10/pR-Pcad-Pmer-G could detect as low as 0.0098 μM Hg(II) with green fluorescence as the output signal (Figure 3C). Furthermore, The detection limit for Hg(II) was improved to 0.3125 μM using both single-sensing TOP10/pR-Pcad and dual-sensing TOP10/pR-Pcad-Pmer-G with red fluorescence as the output signal (Figure 3F). The green fluorescence responsive to Hg(II) always increased upon exposure to Hg(II) below 10 μM, and the RFU of single-sensing TOP10/pPmer-G was significantly higher than that of dual-sensing TOP10/pR-Pcad-Pmer-G (Figure 3D). The concentration of Hg(II) and the RFU of eGFP in single-sensing TOP10/pPmer-G could be well-fitted by a non-linear regression relation in a narrow and low concentration range, and the regression equation and the coefficient of determination (R^2^ = 0.99947) were shown in Figure 3E. The red fluorescence increased upon exposure to Hg(II) below 20 μM in signal-sensing TOP10/pR-Pcad. Then, the red fluorescence sharply decreased due to the high toxicity of 40 μM Hg(II) (Figure 3G). Therefore, the significant increased OD_600_ value in TOP10/pR-Pcad culture exposed to 20 μM Hg(II) (Figure 3A) could be attributed to the high expression of mCherry. Interesting, the RFU of mCherry in dual-sensing TOP10/pR-Pcad-Pmer-G was not further increased upon exposure to Hg(II) upon 2.5 μM (Figure 3G). The Hg(II) concentration and the RFU of mCherry in single-sensing TOP10/pR-Pcad were also well-fitted by a non-linear regression relation in a wide and high concentration range. The regression equation and the coefficient of determination (R^2^ = 0.99807) are shown in Figure 3H.

To confirm the double-color fluorescence of dual-sensing biosensor upon exposure to Hg(II), dual-sensing TOP10/pR-Pcad-Pmer-G was induced with 20 μM Hg(II), and TOP10/pR-Pcad-Pmer-G treated with 0 μM Hg(II) was used as the control group. After culturing at 37*^o^*C for 8 h, biosensor cells were harvested and spread onto the slides. Bright-field and double-fluorescent field images were captured (Supplementary Figure 4). Strong double-color fluorescent signals were detected from biosensor cells treated with 20 μM Hg(II). Very weak double-color fluorescence was observed in the control group. Double-color fluorescent indication for Hg(II) was finally validated in the test.

### Responses of Whole-Cell Biosensors Toward Cd (II)

**FIGURE 4:**
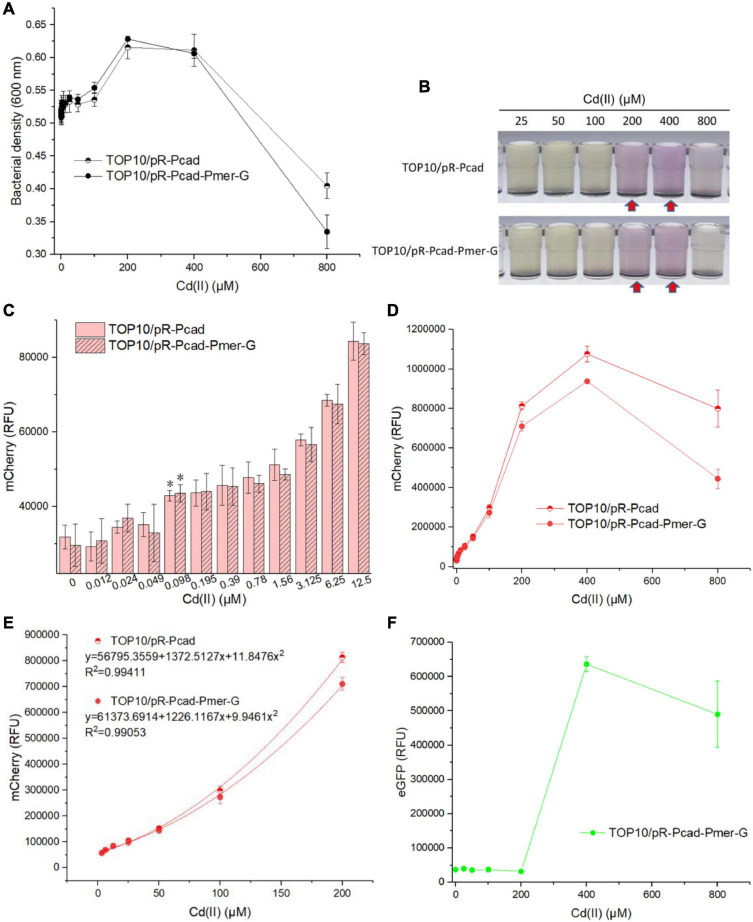
Performance of single-sensing and dual-sensing whole-cell biosensors exposed to increased concentrations of Cd(II). Single-sensing TOP10/pR-Pcad and dual-sensing TOP10/pR-Pcad-Pmer-G in lag phase were exposed to increased concentrations of Cd(II). After incubation at 37*^o^*C for 8 h, bacterial cell densities **(A)** were determined immediately. A representative photo of induced culture **(B)** was shown, and the culture with high absorbance at 600 nm was marked with the red arrows. **(C)** The detection sensitivity with red fluorescence as signal output. The asterisk represents a statistically significant difference (two-tailed *t*-test, *P* < 0.05) compared with the same engineered biosensor with no Cd(II) exposure. **(D)** The dose-dependent red fluorescent response curve with Cd(II) ranges from 0 to 800 μM. **(E)** Regression analysis of the relationship between relative red fluorescent intensity and Cd(II) concentration. **(F)** The dose-dependent green fluorescent response curve with Cd(II) ranges from 0 to 800 μM. Data represent the mean ± SD of at least three independent experiments.

Single-sensing TOP10/pR-Pcad and dual-sensing TOP10/pR-Pcad-Pmer-G were exposed to 0, 0.012, 0.024, 0.049, 0.098, 0.195, 0.39, 0.78, 1.56, 3.125, 6.25, 12.5, 25, 50, 100, 200, 400, and 800 μM Cd(II). Bacterial density and double-color fluorescence were analyzed after culturing at 37*^o^*C for 8 h. Owing to significant cytotoxicity of Cd(II), bacterial densities of two whole-cell biosensors dramatically decreased upon exposure to above 400 μM Cd(II) exposure (Figure 4A). Overexpressed mCherry made the Cd(II)-inducible culture color red at 200 and 400 μM (Figure 4B), and it might enable an increased OD_600_ when exposed to these two concentrations of Cd(II) (Figure 4A). Both single-sensing TOP10/pR-Pcad and dual-sensing TOP10/pR-Pcad-Pmer-G responded to as low as 0.098 μM Cd(II) (Figure 4C). The red fluorescent signals continuously increased when two biosensors were exposed to below 400 μM Cd(II), followed by a significant decrease probably due to the obvious cytotoxicity (Figure 4D). Compared with the red fluorescence of dual-sensing TOP10/pR-Pcad-Pmer-G, that of single-sensing TOP10/pR-Pcad upon exposure to above 200 μM Cd(II) was significantly stronger (Figure 4D). Importantly, the concentration of Cd(II) and the RFU of mCherry in both single-sensing TOP10/pR-Pcad and dual-sensing TOP10/pR-Pcad-Pmer-G were well-fitted by a non-linear regression relation in a wide and high concentration range, and the regression equations and the coefficients of determination were shown in Figure 4E. Interestingly, a strong green fluorescent signal responsive to above 200 μM Cd(II) was also observed in dual-sensing TOP10/pR-Pcad-Pmer-G (Figure 4F).

### Simultaneous Detection of Hg(II) and Cd(II) Using Dual-Sensing Bioreporters

**FIGURE 5:**
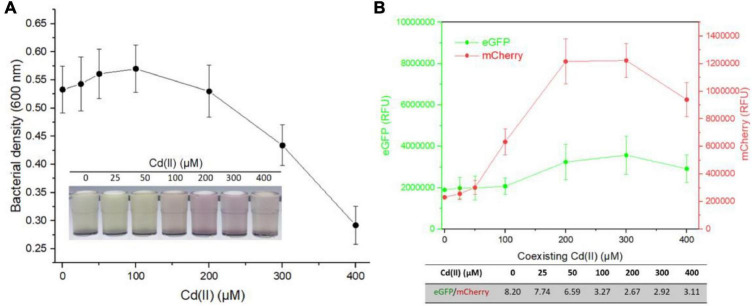
Influence of Hg(II) at a constant concentration on the response of dual-sensing biosensor toward Cd(II) at varied concentrations from 0 to 400 μM. Dual-sensing TOP10/pR-Pcad-Pmer-G in lag phase were exposed to 2.5 μM Hg(II) in the presence of increased concentrations of Cd(II). After incubation at 37*^o^*C for 8 h, bacterial cell densities **(A)** were determined immediately. The inset is a representative photo of induced cultures. Double-color fluorescence derived from TOP10/pR-Pcad-Pmer-G was then determined **(B)**, and the mean of fluorescence intensity ratios (eGFP/mCherry) were shown in the table below the corresponding figure. Data represent the mean ± SD of at least three independent experiments.

To further evaluate the performance of dual-sensing TOP10/pR-Pcad-Pmer-G upon exposure to Hg(II) and Cd(II) simultaneously, TOP10/pR-Pcad-Pmer-G was exposed to 2.5 μM Hg(II) in combination with 0, 25, 50, 100, 200, 300, or 400 μM Cd(II). Bacterial density (Figure 5A) and double-color fluorescence (Figure 5B) were measured after culturing at 37*^o^*C for 8 h. Bacterial cell density showed a downward trend accompanied by an increased concentration of Cd(II) (Figure 5A). A significant rise in red fluorescence accompanied by 0–200 μM Cd(II) was observed. The intensity of red fluorescence remained stable at 200–400 μM Cd(II) and decreased at above 400 μM Cd(II) (Figure 5B). However, the green fluorescence just increased slightly at above 100 μM Cd(II), and it finally led to the significantly decreased ratios of eGFP/mCherry at concentrations of 0–200 μM Cd(II) (Figure 5B).

**FIGURE 6:**
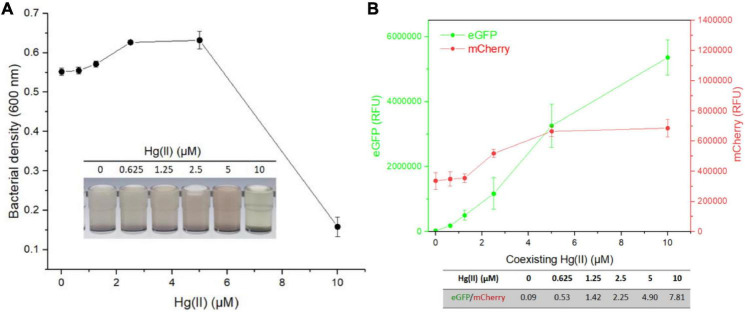
Influence of Cd(II) at a constant concentration on the response of dual-sensing biosensor toward Hg(II) at varied concentrations from 0 to 10 μM. Dual-sensing TOP10/pR-Pcad-Pmer-G in lag phase were exposed to 100 μM Cd(II) in the presence of increased concentrations of Hg(II). After incubation at 37*^o^*C for 8 h, bacterial cell densities **(A)** were determined immediately. The inset is a representative photo of induced cultures. Double-color fluorescence derived from TOP10/pR-Pcad-Pmer-G was then determined **(B)**, and the mean of fluorescence intensity ratios (eGFP/mCherry) were shown in the table below the corresponding figure. Data represent the mean ± SD of at least three independent experiments.

Then, TOP10/pR-Pcad-Pmer-G was exposed to 100 μM Cd(II) in combination with 0, 0.625, 1.25, 2.5, 5, or 10 μM Hg(II). After incubation at 37*^o^*C for 8 h, bacterial density (Figure 6A) and double-color fluorescence (Figure 6B) were analyzed. Due to the increased cytotoxicity of heavy metal, a significant decline in bacterial cell density was observed at above 5 μM Hg(II) in combination with 100 μM Cd(II) (Figure 6A). A significant rise in green fluorescence accompanied with 0–10 μM Hg(II) was observed. However, just a slight rise in red fluorescence was found at above 1.25 μM Hg(II), and it undoubtedly led to the significantly enhanced ratios of eGFP/mCherry at concentrations of 0–10 μM Hg(II) (Figure 6B).

## Discussion

In most MerR-family operons, metalloregulators are divergently located to a series of heavy metal resistant genes which form an operon. Transcription of these resistant genes is repressed in the absence of cognate metal ions and activated in the presence of cognate metal ions (Borremans et al., 2001; Lee et al., 2001; Mathema et al., 2011). However, it is not well-known whether the transcriptional level of the metalloregulator is upregulated or not when a bacterium is exposed to the cognate metal ion. As shown in Figure 1A, the *rrn*B terminator has been placed downstream of the fluorescent reporters. A DNA hairpin will form if another *rrn*B terminator is positioned downstream of the metalloregulator, and the form of DNA hairpin usually leads to the failure of molecular cloning. Our finding showed that the expression of fluorescent reporter located downstream of MerR or CadR was not significantly enhanced upon exposure to increased concentrations of Hg(II) or Cd(II) (Supplementary Figure 2), suggesting that the basal expression of MerR or CadR in these genetic constructs is nearly constant.

Furthermore, the reversed insertion of the sensory module exerted no significant effects on the production of biosensing signal (Supplementary Figure 3). Therefore, the assembly strategy of the dual-sensing construct is chosen, as shown in Figure 1B. Double-color fluorescent signals are expected to be differentially emitted without mutual interference.

Compared with previous studies (Wang et al., 2020; Zhang et al., 2021), the single-sensing TOP10/pPmer-G was also demonstrated to respond to Hg(II) selectively. However, an increased response toward 250 μM Cd(II) was firstly found in this study (Figure 2A). Previously developed CadR-based whole-cell biosensors using various genetic circuit designs were found to respond to Cd(II) and Hg(II) (Bereza-Malcolm et al., 2017; Guo et al., 2021b; Hui et al., 2021c). As expected, the single-sensing TOP10/pR-Pcad responded strongly to Cd(II) and Hg(II) (Figure 2B). The biosensing characteristics of two single-sensing constructs were integrated into the dual-sensing TOP10/pR-Pcad-Pmer-G. Green fluorescence specifically toward Hg(II) (Figure 2C) and red fluorescence toward Cd(II) and Hg(II) (Figure 2D) were found in the selectivity assay. Only a red fluorescent signal was induced when dual-sensing TOP10/pR-Pcad-Pmer-G was exposed to 5, 25, and 125 μM Cd(II) (Figure 2D), and thus the intensity of red fluorescence was similar to that of single-sensing TOP10/pR-Pcad (Figure 2B). Upon exposure to 250 μM Cd(II), a slightly enhanced green fluorescence was induced in TOP10/pR-Pcad-Pmer-G culture (Figure 2C). It led to a slightly decreased red fluorescence toward 250 μM Cd(II) (Figure 2D). Compared with single-signal output, multiple-signal output often resulted in the biosensing signal attenuation, which is attributed to increased metabolic burden and excessive energy consumption in a single sensory cell (Guo et al., 2021b; Hui et al., 2021c; Zhang et al., 2021). This phenomenon is evident when dual-sensing TOP10/pR-Pcad-Pmer-G was exposed to Hg(II). Compared with the green fluorescence emitted by single-sensing TOP10/pPmer-G (Figure 2A) and the red fluorescence emitted by single-sensing TOP10/pR-Pcad (Figure 2B), Hg(II)-inducible double-color fluorescent signals significantly decreased in TOP10/pR-Pcad-Pmer-G culture (Figures 2C,D).

As shown in Figure 2, bioavailable Hg(II) can induce the expression of eGFP and mCherry, which is derived from the *merR*-P*mer-egfp* genetic cassette and the *cadR*-P*cad-mcherry* genetic cassette, respectively. Double-color fluorescent signals responsive to Hg(II) made the response of dual-sensing TOP10/pR-Pcad-Pmer-G toward Hg(II) is complex. Overexpressed fluorescence protein made the culture color (Figure 3B), which further led to a corresponding increased bacterial density when measured at 600 nm (Figure 3A). The OD_600_ value cannot precisely reflect bacterial cell density when the culture is colored (Hui et al., 2021c; Zhang et al., 2021). Due to the attenuation of biosensing signals resulting from the double-fluorescence output (Guo et al., 2021b; Zhang et al., 2021), the detection limit of green fluorescence derived from the *merR*-P*mer-egfp* module was slightly increased (Figure 3C) and further led to a significantly decreased response to increased concentrations of Hg(II) (Figure 3D). Although the detection limit of red fluorescence derived from the *cadR*-P*cad-mcherry* module was not influenced (Figure 3F), the red fluorescence toward Hg(II) was considerably changed due to the dual-sensing design. We can conclude that two single-sensing biosensors are more suitable for determining bioavailable Hg(II) than a dual-sensing biosensor. After all, the dual-sensing biosensor was initially designed to find use in detecting samples mixed with Cd(II) and Hg(II).

Owing to the silent response of the *merR*-P*mer-egfp* module toward Cd(II) below 200 μM, bacterial density, the detection limit, the dose-response curve, and the non-linear regression relation were similar between single-sensing TOP10/pR-Pcad and dual-sensing TOP10/pR-Pcad-Pmer-G upon exposure to Cd(II) bellow 200 μM (Figure 4). Furthermore, a strong red fluorescence (700,000 cnt above) accompanied with a strong green fluorescence (below 700,000 cnt) will suggest the sample contains 200 μM above Cd(II) when the dual-sensing biosensor is used. Most importantly, the silent green fluorescent signal usually shows no bioavailable Hg(II) in the sample, and the content of Cd(II) can be roughly quantitated using red fluorescence. However, we usually cannot tell whether Cd(II) or Hg(II) induces the red fluorescence when the single-sensing TOP10/pR-Pcad is used (Guo et al., 2021b).

Multiple heavy metal pollutants usually coexist in a natural ecosystem, and it promotes the development of biosensors differentially responding to various heavy metals (Bereza-Malcolm et al., 2015; Hou et al., 2015; Yoon et al., 2016). However, an excellent anti-jamming capability is crucial for a multiple-sensing biosensor. Most MerR family metalloregulators selectively respond to their cognate metal ions, and these regulators are ideal sensory elements to develop biosensors (Jung and Lee, 2019). Compared with the developed high selective whole-cell biosensors toward Pb(II) (Wei et al., 2014; Bereza-Malcolm et al., 2016; Guo et al., 2019) and Hg(II) (Cai et al., 2018; Wang et al., 2020), the specificity of currently available whole-cell biosensors toward Cd(II) is unsatisfactory (Kumar et al., 2017; Jia et al., 2021). Our previous studies showed that integrating two kinds of Cd(II) sensory elements into one biosensing construct contributed to the improved selective response to Cd(II) and Pb(II), but not to Hg(II) (Hui et al., 2021c). This finding prompted the design of a dual-sensing biosensor with the combination of Hg(II) sensory element with Cd(II) sensory component of this study. When this developed dual-sensing biosensor was exposed to a mixture containing Cd(II) and Hg(II), significantly stronger red fluorescence reflected the higher content of Cd(II) (Figure 5). In contrast, significantly stronger green fluorescence reflected the higher concentration of Hg(II) (Figure 6).

Our novel dual-sensing biosensor has proven to be efficacious in identifying mono-contamination by bioavailable Cd(II). Although this dual-sensing biosensor cannot precisely distinguish Cd(II) from Hg(II) in a sample with two co-existent heavy metals, it does help identify the dominant heavy metal. This study suggests that the combined employment of several sensory elements improves the specificity of the resultant biosensor.

## Data Availability Statement

The original contributions presented in the study are included in the article/Supplementary Material, further inquiries can be directed to the corresponding author.

## Author Contributions

C-YH designed the experimental protocol and drafted the manuscript. YG, HL, and Y-TC carried out the majority of the study. YG and JY analyzed the data. All authors read and approved the submitted version.

## Conflict of Interest

The authors declare that the research was conducted in the absence of any commercial or financial relationships that could be construed as a potential conflict of interest.

## Publisher’s Note

All claims expressed in this article are solely those of the authors and do not necessarily represent those of their affiliated organizations, or those of the publisher, the editors and the reviewers. Any product that may be evaluated in this article, or claim that may be made by its manufacturer, is not guaranteed or endorsed by the publisher.
