# Supplementary material for: Differential Detection of Bioavailable Mercury and Cadmium Based on a Robust Dual-Sensing Bacterial Biosensor
Source: Front Microbiol. 2022 Apr 13;13:846524. doi: 10.3389/fmicb.2022.846524 (PMC9043898; doi:10.3389/fmicb.2022.846524)
Supplement: Supplementary file 1 [file Data_Sheet_1.docx]

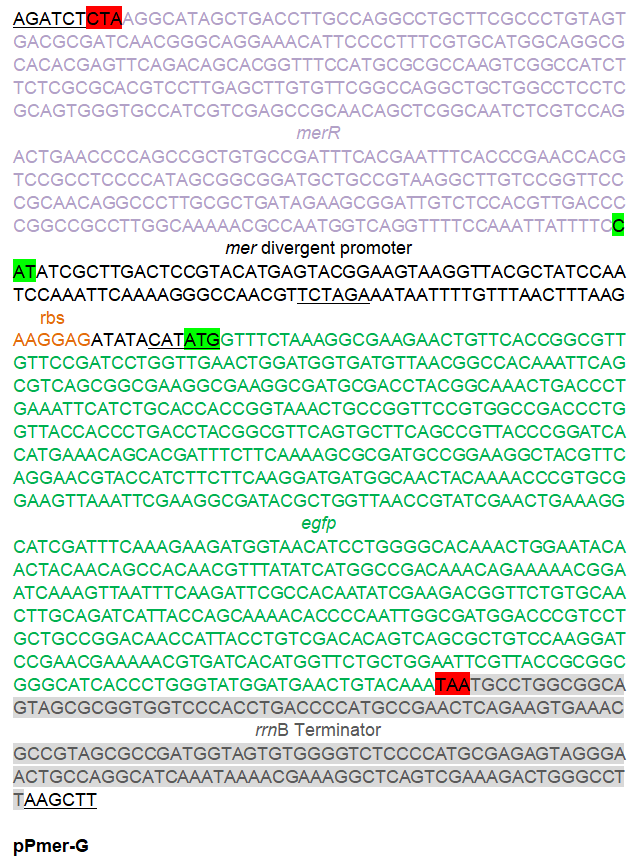


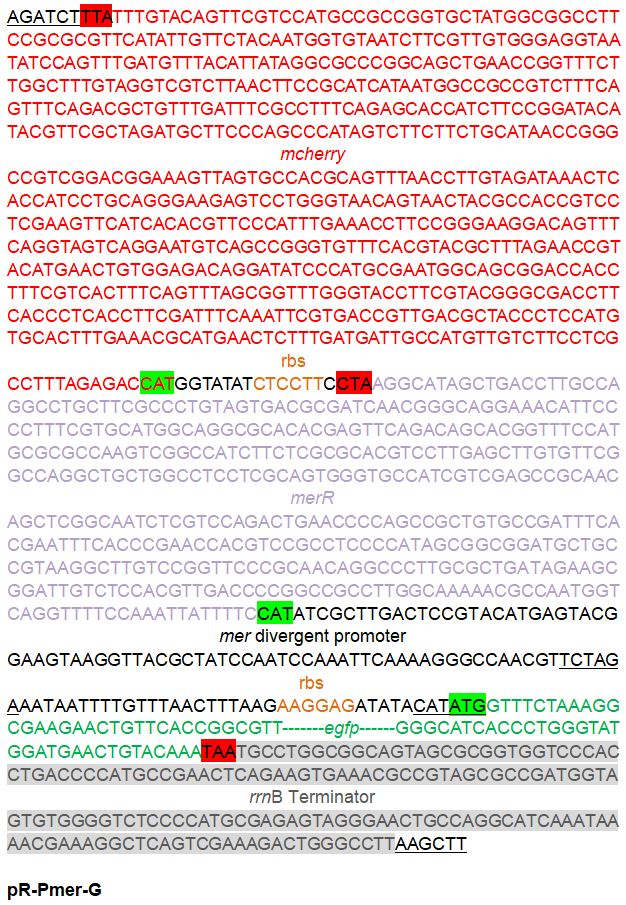


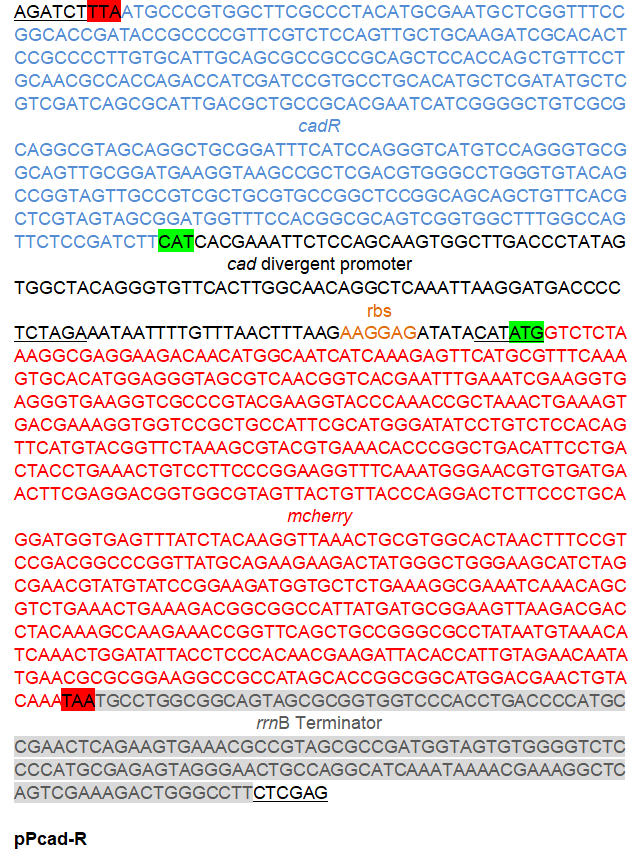


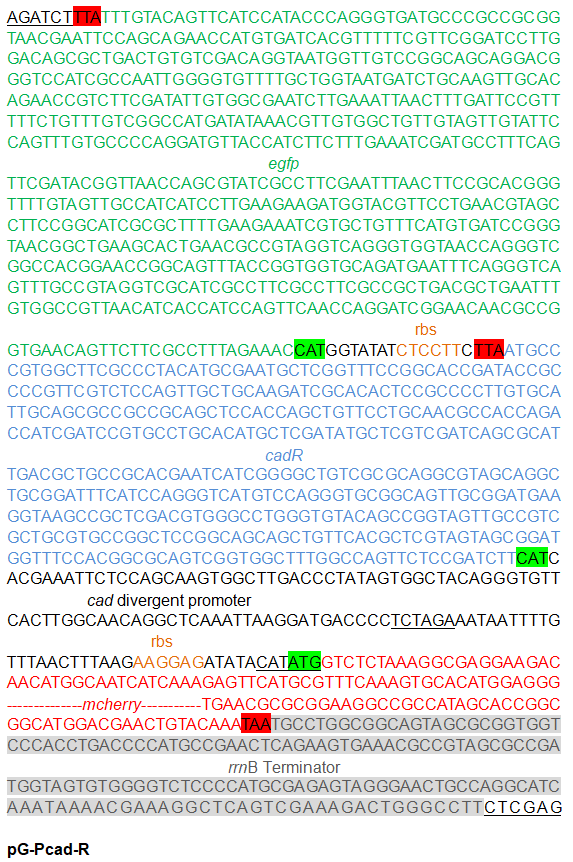


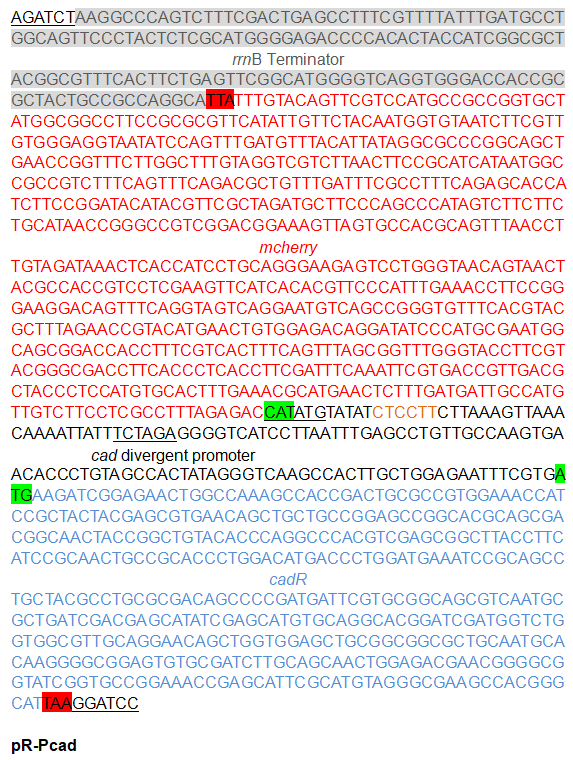


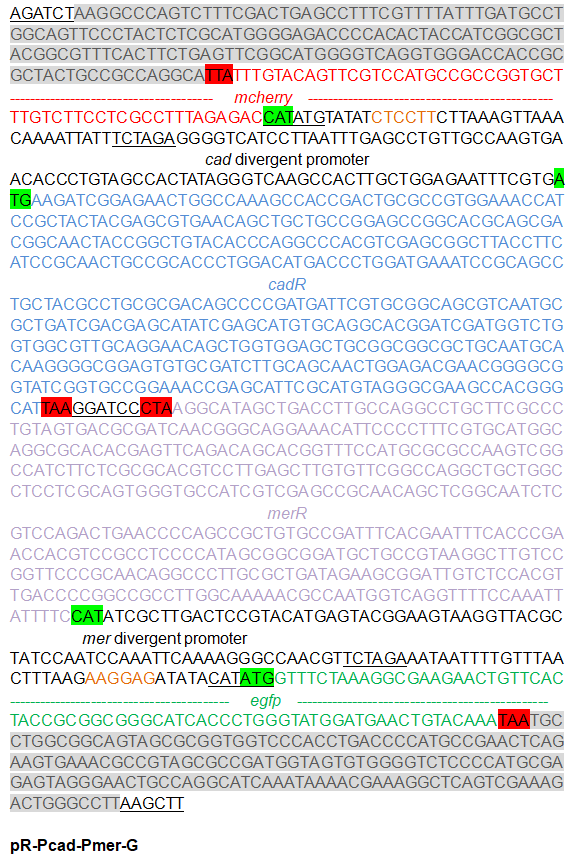


**Fig. S1** The cloning/expression region of recombinant plasmids used in this study. DNA sequence and annotation data are all marked.


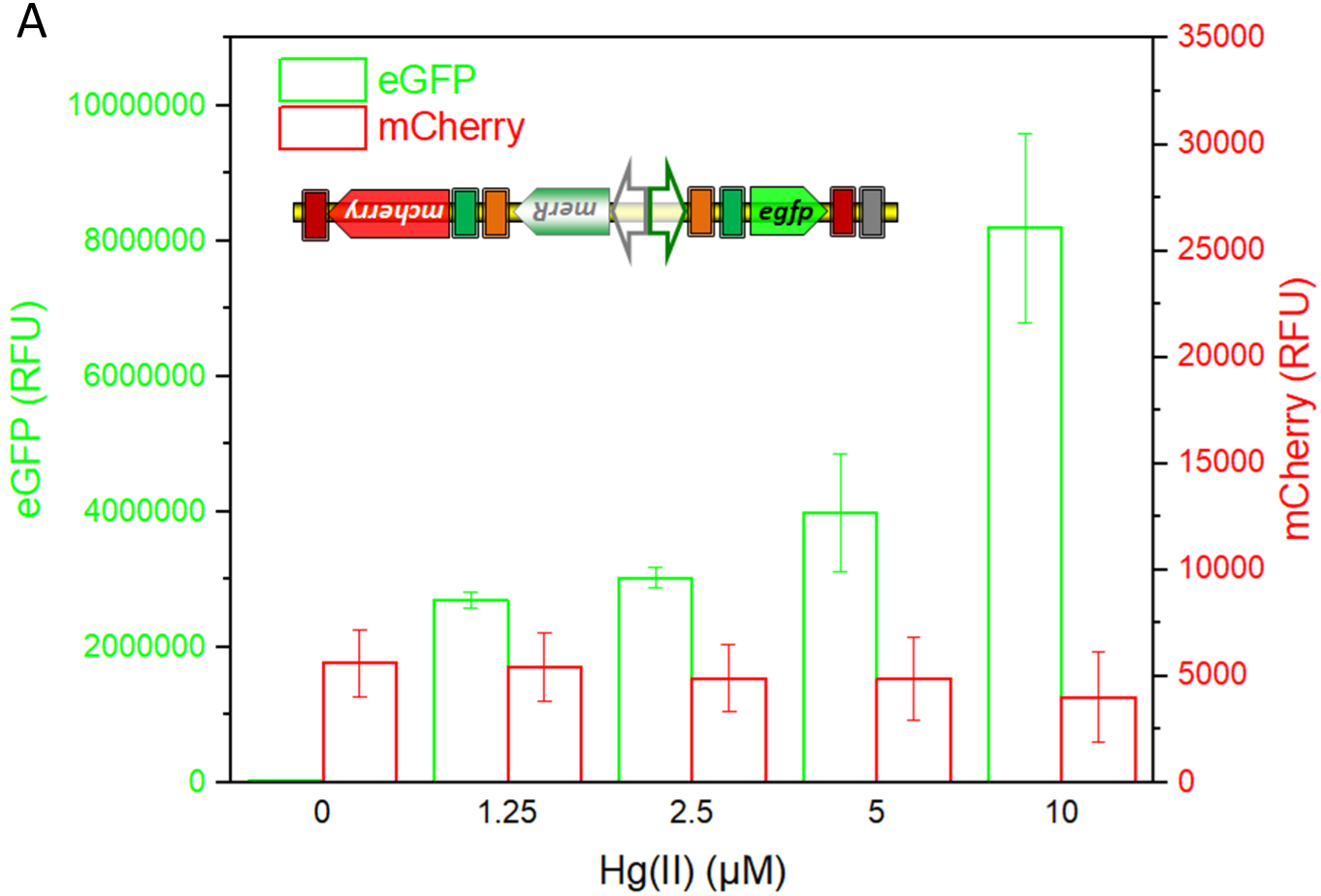

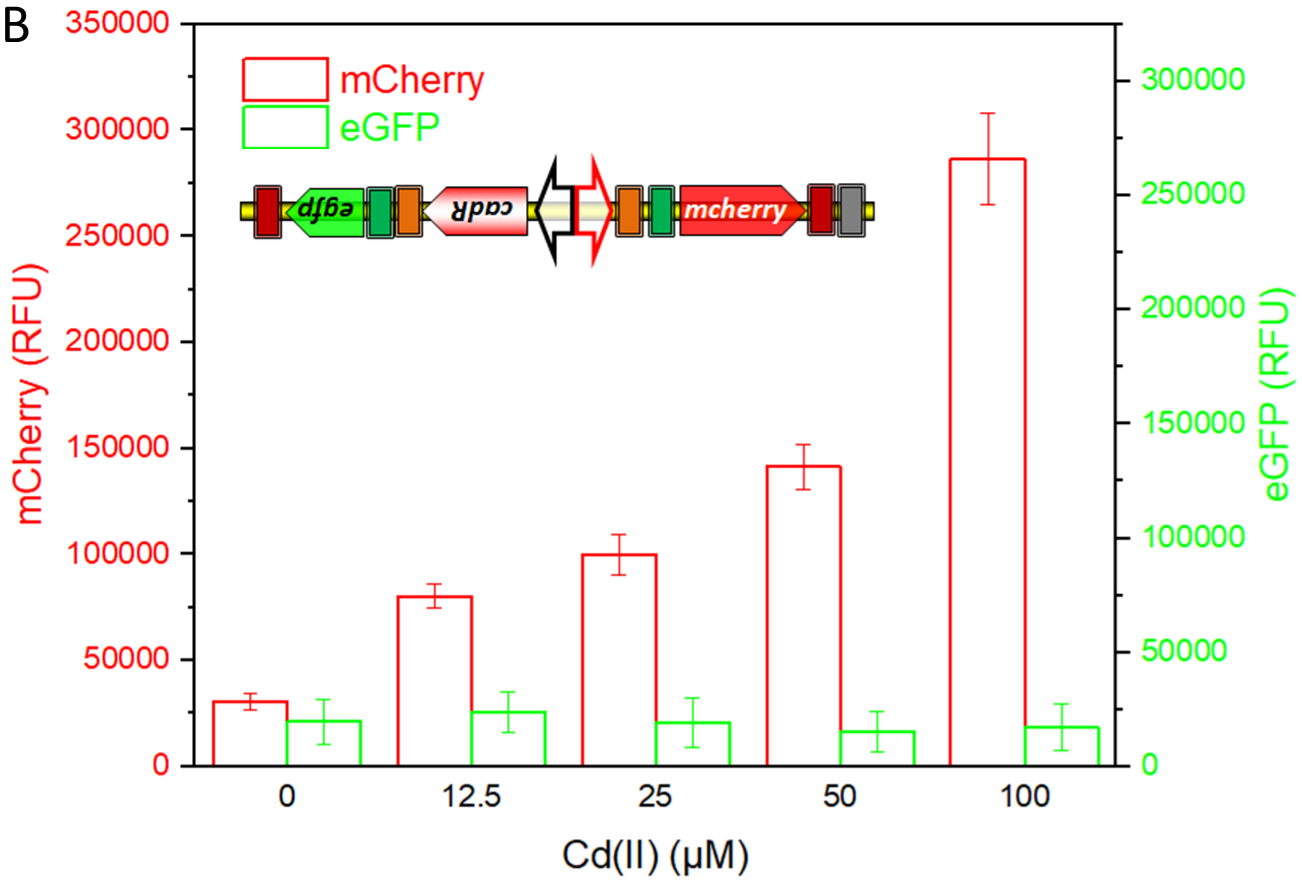


**Fig. S2** Bidirectional responses of single-sensing biosensors toward their cognate metal ions.

Whole-cell biosensor TOP10/pR-Pmer-G in lag phase was exposed to increased concentrations of Hg(II) (A), and TOP10/pG-Pcad-R in lag phase was exposed to increased concentrations of Cd(II) (B). After incubation at 37 ^o^C for 8 h, double-color fluorescent signals were determined. Fluorescence intensity values were divided by the absorbance at 600 nm in order to normalize to bacterial cell concentration. Data are expressed as the mean ± SD from at least three independent experiments.


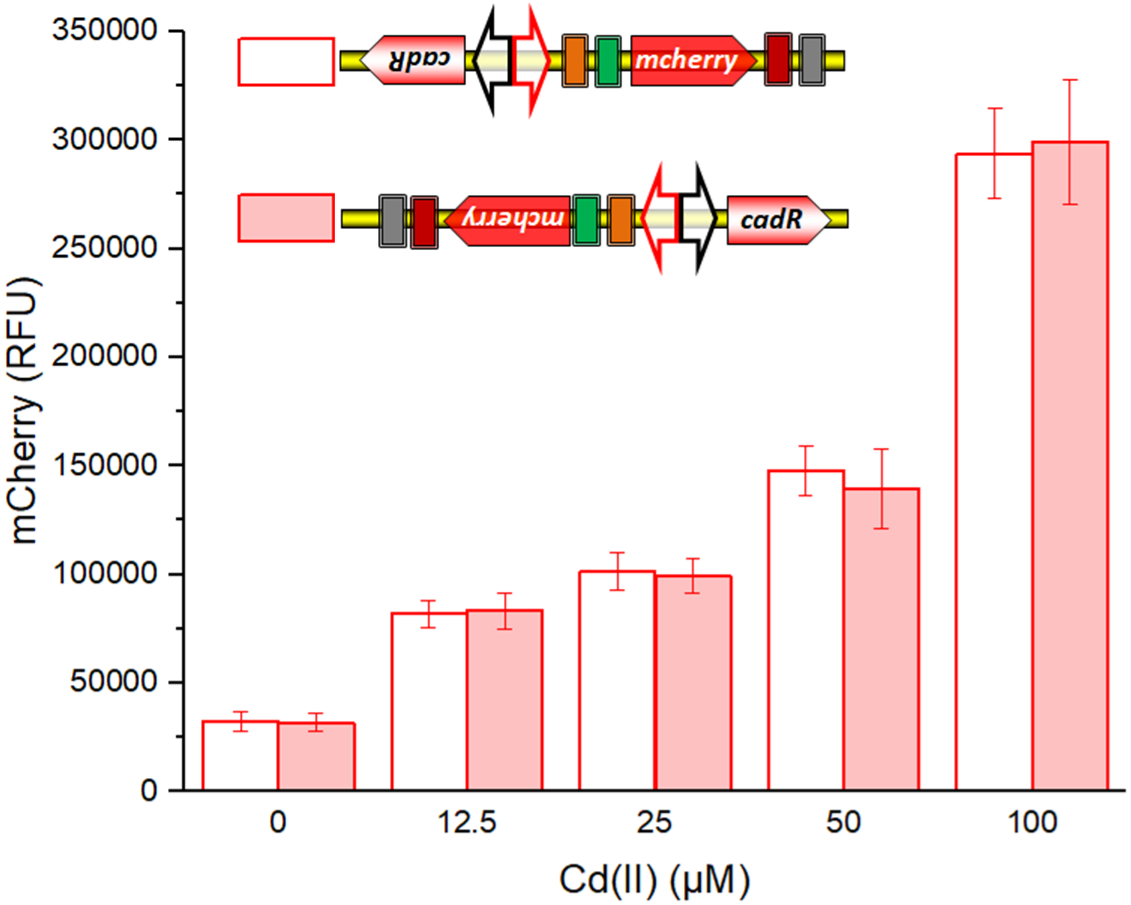


**Fig. S3** Comparison of reporter signals generated from whole-cell biosensors with inverted genetic assembly.

Whole-cell biosensors TOP10/pPcad-R and TOP10/pR-Pcad in lag phase were exposed to gradient concentrations of Cd(II). After incubation at 37 ^o^C for 8 h, red fluorescent signal derived from Cd(II)-inducible expressed mCherry was determined. Fluorescence intensity values were normalized using the absorbance at 600 nm. Data represent the mean ± SD of at least three independent experiments.


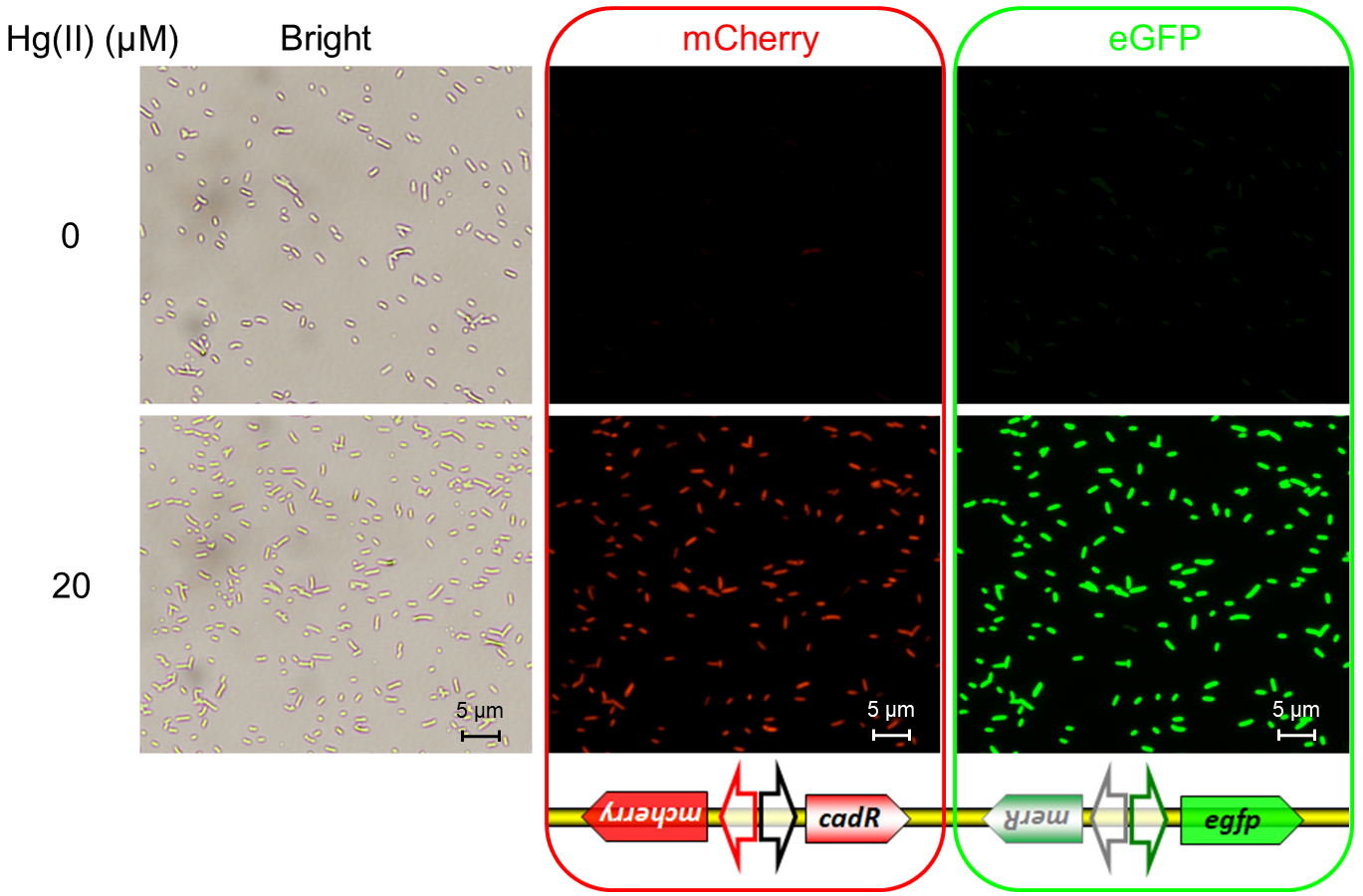


**Fig. S4** Fluorescence responses of dual-sensing TOP10/pR-Pcad-Pmer-G upon exposure to Hg(II).

Dual-sensing TOP10/pR-Pcad-Pmer-G in lag phase were exposed to 0 μM or 20 μM Hg(II). After incubation at 37 ^o^C for 8 h, bacterial cells were visualized using a fluorescence microscope (×400 magnification) equipped with a Texas Red filter for mCherry image and a FITC filter for eGFP image. Shown are representative images from three independent assays.
